# Long Non-coding RNAs as Communicators and Mediators Between the Tumor Microenvironment and Cancer Cells

**DOI:** 10.3389/fonc.2019.00739

**Published:** 2019-08-06

**Authors:** Di Chen, Tong Lu, Junying Tan, Hao Li, Qiuyue Wang, Liangzhou Wei

**Affiliations:** ^1^Department of Gastroenterology, Affiliated Hospital of Qingdao University, Qingdao, China; ^2^Department of Thoracic Surgery, Affiliated Hospital of Qingdao University, Qingdao, China

**Keywords:** long non-coding RNA, tumor microenvironment, stromal cells, exosomes, therapy

## Abstract

Long non-coding RNAs (lncRNAs) are a class of more than 200 nucleotides RNA transcripts which have limited protein coding capacity. They regulate numerous biological processes in cancers through diverse molecular mechanisms. Aberrant expression of lncRNAs has been frequently associated with human cancer. Furthermore, the tumor microenvironment (TME) is composed of different cells such as cancer-associated fibroblasts (CAFs), endothelial cells and infiltrated immune cells, and all of which participate in communication with tumor cells affecting the progression of tumor. LncRNAs are directly and indirectly involved in the crosstalk between stromal cells and tumor cells and dysregulated lncRNAs expression in these cells could drive tumorigenesis. In this review, we explore the influence of aberrantly expressed lncRNAs in tumor progression, clarify the critical roles of lncRNAs in the TME, summarize findings on crosstalk between infiltrated immune cells, CAFs, endothelial cells, and tumor cells via lncRNAs, and discuss the promise of lncRNAs as tumor diagnostic markers and therapeutic targets.

## Introduction

Long non-coding RNAs (lncRNAs) are a diverse class of transcribed RNA molecules that are more than 200 nucleotides with limited protein coding potential (1, 2). Current estimates, from the GENCODE database (www.gencodegenes.org), indicate that the human genome contains ~16,000 lncRNA genes that encode more than 28,000 distinct lncRNAs. Many lncRNAs have emerged as critical players in regulating numerous biological processes in cancer, such as differentiation, cell cycle regulation, and immune response (3–5). They can directly act as tumor suppressors or oncogenes, or are regulated by well-known tumor suppressors or oncogenes, at transcriptional or post-transcriptional levels (6, 7). Furthermore, emerging evidence have shown that dysregulated lncRNAs are greatly involved in various cancers (8–10). For instance, the PCA3 (also called DD3) and PCGEM1 were the first lncRNAs that were associated with cancer because they were overexpressed in prostate cancer (11, 12). PCA3 might be used as a biomarker for the diagnosis of prostate cancer and PCGEM1 involved in c-MYC activation and androgen receptor transcriptional activation was associated with the progression of prostate cancer (13, 14). Moreover, lncRNA MALAT1 was reported to be overexpressed in multiple cancer types, such as colorectal cancer, non-small cell lung cancer and hepatocellular carcinoma (HCC); its expression was also correlated with tumor progression and poor prognosis (15–18). Importantly, these studies suggested that aberrantly expressed lncRNAs can be used as biomarkers for cancer diagnosis and prognosis as well as potential targets for cancer therapy.

The tumor microenvironment (TME) is a complicated physiological and biochemical system that plays critical functions in tumorigenesis, progression, and metastasis (19–21). In addition to tumor cells, the TME consists mainly of the extracellular matrix (ECM), the tumor vascular system, other non-malignant cells as well as the acidic and hypoxic environment of the tumor (21, 22). With the development of biological technology, different cell types have been identified in the TME, including cancer-associated fibroblasts (CAFs), fat cells, endothelial cells, and infiltrated immune cells such as T lymphocytes, myeloid-derived suppressor cells, and tumor-associated macrophages (22–24). Most of these stromal cells significantly contribute to the initiation and progression of tumors. In recent years, a growing appreciation of the TME indicated that lncRNAs play significant roles in the interactions between tumor cells and stromal cells (25–30). In this review, we discuss the role of lncRNAs in the crosstalk between CAFs, endothelial cells, infiltrated immune cells and tumor cells, and the promise of lncRNAs in cancer treatment, to increase our knowledge of the function of lncRNAs within the TME and lay a foundation for lncRNA-based anti-tumor treatment strategies.

## LncRNAs and CAFs

CAFs, one of the most abundant stromal cells in the TME, are critically involved in tumor progression (31, 32). CAFs modulate the biology of cancer cells through releasing numerous regulatory factors, such as chemokines, cytokines and growth factors, and thus these cells affect the progression of tumor (31, 33). Transforming growth factor-β1 (TGF-β1), which is secreted by CAFs, is a critical factor promoting the epithelial-mesenchymal transition (EMT) and metastasis of cancer cells such as bladder cancer cells and breast cancer cells (34, 35). And the secretion of TGF-β1 by CAFs induces the metastatic activity of cancer cells by regulating the expression of lncRNAs. For instance, CAFs promoted EMT of bladder cancer cells by activating the transcription of lncRNA-ZEB2NAT via TGF-β1 secretion (34). Similarly, TGF-β1 secreted by CAFs upregualted lncRNA HOTAIR expression to promote EMT and metastasis in breast cancer (35). Furthermore, in oral squamous cell carcinoma, LncRNA-CAF could elevate the expression of cytokine IL-33 to promote the activation of CAFs, leading to proliferation of tumor cells. In return, tumor cells also secreted exosomes including LncRNA-CAF to stroma and increased LncRNA-CAF levels for the activation of CAFs (36).In addition, LINC00092 was found to be upregulated in response to the CAF-secreted chemokine CXCL14 in ovarian cancer cells. Simultaneously, LINC00092, which induced a glycolytic phenotype of ovarian cancer cells, was critical for the maintenance of CAF-like features by interacting with PFKFB2 (37). Therefore, LncRNA-CAF and LINC00092 were served as significant modulators of feedback loop in the cancer cells and CAFs, which were critical for the progression of cancer. Collectively, these studies indicated the importance of lncRNAs in the interaction between the CAFs and cancer cells and provided a potential application for lncRNAs as targets of cancer treatment.

## LncRNAs and Endothelial Cells

Endothelial cells, which line the interior surface of blood vessels, are important components of stroma in TME (26, 38). They are believed to be critical for angiogenesis and tumor metastasis, and lncRNAs may affect the progression of tumor through modulating the biological behavior of endothelial cells. LncRNA H19, for instance, was reported to be significantly upregulated in glioma-associated endothelial cells cultured in glioma-conditioned medium. Knockdown of H19 inhibited glioma-induced endothelial cell proliferation, migration and tube formation *in vitro*. Mechanistic evidence revealed that H19 modulated the biological behavior of glioma-associated endothelial cells by suppressing miR-29a (39). Furthermore, lncRNA-APC1 played an important tumor-suppressive role in the pathogenesis of colorectal carcinoma. Following mechanism studies showed that lncRNA-APC1 decreased exosome production in colorectal carcinoma cells through reducing the stability of Rab5b mRNA, and this action suppressed tumor angiogenesis through inhibiting the overactivation of the MAPK pathway in endothelial cells (40). In summary, dysregulated lncRNAs affect the biological behavior of endothelial cells by diverse mechanisms, thus modulating specific lncRNA expression in tumor cells or/and endothelial cells may have a significant effect on the progression of cancer.

## LncRNAs Involved in Crosstalk Between Infiltrated Immune Cells and Tumor Cells

### Tumor-Associated Macrophages (TAMs)

TAMs are important regulators of the TME, and might regulate tumor growth, invasion, and metastasis (41). Two major functional types of macrophages have been identified, including classically activated (M1) and alternatively activated (M2) macrophages (23, 41, 42). M1 macrophages participate in the Th1-type inflammatory response and have anti-tumorigenic functions, while M2 macrophages promote anti-inflammatory responses and have a pro-tumorigenic role (23, 43, 44). Several studies have indicated that lncRNAs could modulate M2 polarization of macrophages to affect tumor cells migration and invasion. For example, lncRNA CCAT1 could modulate the TME of prostate cancer through regulating macrophages polarization. Knockdown of lncRNA CCAT1 enhanced macrophages polarization to M2 through upregulating the expression of miR-148a and further promoted prostate cancer cells migration and invasion (45). LncRNA NIFK-AS1 also played a key role in modulating the polarization of TAMs in endometrial cancer. It could suppress M2 macrophages polarization by inhibiting miR-146a, thus reducing the endometrial cancer cells proliferation, migration and invasion (46). Moreover, a cell model-based microarray analysis was used to detect lncRNAs involved in M2 polarization and lncRNA-MM2P was found to be upregulated during M2 polarization. In addition, further studies demonstrated that lncRNA-MM2P promoted M2 polarization of macrophages by reducing phosphorylation on STAT6, then affecting macrophage-mediated tumorigenesis and tumor growth (47).

Furthermore, CCL2, which is produced by different tumor types, plays a critical role in tumor metastasis (48, 49). Tumor-derived CCL2 is released into the TME and recruits macrophages to tumor cells, which contribute to tumor cells proliferation, angiogenesis, and immune response evasion (48, 50). It has been demonstrated that lncRNAs could modulate the TME by regulating the expression of CCL2 and further affected metastasis. For example, lncRNA LNMAT1 was greatly upregulated in lymph node-metastatic bladder cancer. The authors showed that LNMAT1 activated the transcription of CCL2 through enhancing hnRNPL-mediated H3K4me3 at the CCL2 promoter. Moreover, LNMAT1-induced CCL2 regulated the TME through recruiting TAMs, ultimately resulting in lymphatic metastasis of bladder cancer (51). Similarly, the expression of lnc-BM was markedly upregulated in breast cancer cells. High expression of lnc-BM also promoted breast cancer brain metastasis in preclinical mouse models. Mechanistically, lnc-BM induced STAT3-dependent expression of CCL2 to attract macrophages to cancer cells, thus enhancing breast cancer brain metastasis (52).

### Myeloid-Derived Suppressor Cells (MDSCs)

MDSCs generated in the bone marrow are one of the major components of TME, and these cells play a critical role in cancer progression by suppressing immune responses (53, 54). MDSCs have immunosuppressive activity in pathological conditions through numerous mechanisms involving inducible NO synthase (iNOS), arginase 1 (ARG1), oxygen species (ROS), and nitric oxide (NO) (54, 55). It has been demonstrated that several lncRNAs such as lnc-chop, lnc-C/EBPβ, and lncRNA Pvt1 contributed to the regulation of the immunosuppressive function of MDSCs through modulating the production of ROS and NO or ROS and ARG1.

Notably, lnc-chop interacted with CHOP and the C/EBPβ isoform LIP to increase the activity of C/EBPβ and upregulate target transcripts, such as ARG1, NOS2, COX2, and NOX2. High levels of these target transcripts could result in the production of ROS and NO, thus promoting tumor growth by enhancing the immunosuppression function of MDSCs (56). In contrast, a recent study found that lnc-C/EBPβinhabited the activation of C/EBPβ, decreased the expression of NO and ROS, and further suppressed the immunosuppressive capacity of MDSCs in the tumor environment (57). Granulocytic MDSCs (G-MDSCs) constitute ~70–80% of MDSCs in cancer patients and tumor-bearing mice (58–60). A recent study showed that LncRNA Pvt1 was critical in modulating the immunosuppressive activity of G-MDSCs. The author found that lncRNA Pvt1 knockdown significantly suppressed G-MDSC-mediated immunosuppression *in vitro* by decreasing the level of ROS and ARG1. In addition, knockdown of lncRNA Pvt1 delayed tumor progression in tumor-bearing mice by inhibiting the function of G-MDSCs (61). These findings suggest that lncRNAs play significant roles in the control of tumor-associated MDSCs and lncRNAs may be potential antitumor immunotherapy targets.

### T Cells

T cells, a predominant immune cell type in the TME, can exert both tumor promoting and suppressive functions, as determined by their effector functions (24, 62). LncRNAs have been gradually recognized as modulators of T cell development, activation and differentiation (63). CD8^+^ T cells, major population of T cells, are prominent anti-tumor cells in TME (60). Recent studies have shown that lncRNAs could modulate the function of CD8^+^ T cells in the TME through diverse mechanisms, further affecting the progression of cancer. Lnc-Tim3 was found to be upregulated and negatively correlated with the production of IL-2 and IFN-γ in tumor-infiltrating CD8^+^ T cells of patients with HCC. Mechanistically, lnc-Tim3 bound to Tim-3 and induced nuclear translocation of Bat3 in HCC, which compromised anti-tumor immunity by promoting CD8 + T cell exhaustion (64). Moreover, lnc-sox5 was significantly upregulated in colorectal cancer tissues and lnc-sox5 knockdown promoted the cytotoxicity and infiltration of CD8^+^T cells. Mechanistic evidence revealed that lnc-sox5 regulated the CD8^+^T cell infiltration and cytotoxicity through modulating the expression of IDO and therefore affecting the progression of colorectal cancer (65). These data indicate that lnc-Tim3 and lnc-sox5 play different roles in modulating the CD8^+^T cells to affect the progression of tumor.

**Figure 1:**
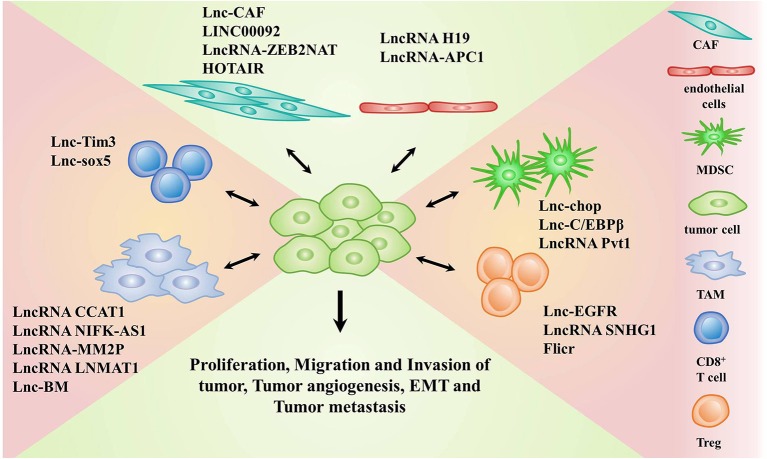
LncRNAs implicated in the crosstalk between stromal cells and tumor cells. Stromal cells include cancer associated fibroblasts (CAFs), endothelial cells, myeloid-derived suppressor cells (MDSCs), tumor-associated macrophages (TAMs), CD8^+^T cells, and regulatory T cells (Tregs).

Regulatory T cells (Tregs) are an immunosuppressive subset of CD4^+^ T cells, which may contribute to the suppression of anti-tumor immunity as they frequently accumulate in the TME (66, 67). Several studies have revealed that some lncRNAs such as lnc-EGFR, lncRNA SNHG1, and Flicr modulated the function of Tregs. Among them, lnc-EGFR was found to be overexpressed in Tregs of patients with HCC. It stimulated the differentiation of Tregs and promoted the growth of tumor in an EGFR-dependent manner. Mechanistic evidence revealed that lnc-EGFR specially bound to EGFR and enhanced AP-1/NF-AT1/Foxp3 signaling, leading to Treg differentiation, and HCC progression (68). LncRNA SNHG1 was also reported to promote the differentiation of Tregs. Knockdown of lncRNA SNHG1 could suppress Treg differentiation by increasing the expression of miR-448 and reducing level of IDO, further alleviating the immune escape in breast cancer (69). Moreover, Flicr, a lncRNA, was reported to enhance the immunosuppressive function of Tregs by modulating the expression of Foxp3. So, Flicr might be associated with tumors, but its roles in the TME remains unclear (70). Collectively, these data indicate that the targeting of specific lncRNAs in T cells is promising for tumor therapy. LncRNAs involved in the communication between tumor cells and stromal cells are shown in Table 1 and Figure 1.

**Table 1:** LncRNAs involved in the crosstalk between stromal cells and tumor cells.

**LncRNA**	**Cancer type**	**Stromal cells**	**Mechanisms of action**	**Reference**
LncRNA-CAF	Oral squamous cell carcinoma	CAFs	Lnc-CAF up-regulates IL-33 expression to reprogram CAFs, promoting tumor development.	(36)
LINC00092	Ovarian cancer	CAFs	CAFs-secreted CXCL14 induces LINC00092 upregulation which promotes ovarian cancer metastasis by enhancing PFKFB-2 translation.	(37)
LncRNA-ZEB2NAT	bladder cancer	CAFs	TGFβ1 secreted by CAFs induces epithelial-mesenchymal transition and invasion of bladder cancer cells via lncRNA-ZEB2NAT.	(34)
HOTAIR	Breast cancer	CAFs	TGFβ1 secreted by CAFs induces epithelial-mesenchymal transition and metastasis of breast cancer through HOTAIR.	(35)
LncRNA H19	Glioma	endothelial cells	Knockdown of H19 suppresses proliferation and migration of glioma-associated endothelial cells through upregulating miR-29a.	(39)
LncRNA-APC1	Colorectal carcinoma	endothelial cells	LncRNA-APC1 suppresses the MAPK pathway overactivation in endothelial cells and further inhibits tumor angiogenesis by increasing exosome production in colorectal carcinoma cells.	(40)
LncRNA CCAT1	Prostate cancer	TAMs	LncRNA CCAT1 knockdown enhances macrophages polarization to M2 and promotes prostate cancer cell migration and invasion through upregulating the expression of miR-148a.	(45)
LncRNA NIFK-AS1	Endometrial cancer	TAMs	LncRNA NIFK-AS1 suppresses the M2 macrophages polarization by inhibiting miR-146a, thus reducing the endometrial cancer cell proliferation, migration and invasion.	(46)
LncRNA-MM2P	–	TAMs	LncRNA-MM2P promotes M2 polarization of macrophages via reducing phosphorylation on STAT6, then affecting macrophage- mediated tumorigenesis and tumor growth.	(47)
LncRNA LNMAT1	Bladder cancer	TAMs	LncRNA LNMAT1-induced CCL2 recruits TAMs, leading to lymphatic metastasis of bladder cancer.	(51)
Lnc-BM	Breast cancer	TAMs	Lnc-BM induces STAT3-dependent expression of CCL2 to attract macrophages, thus enhancing breast cancer brain metastasis.	(52)
Lnc-chop	–	MDSCs	Lnc-chop upregulates the production of ROS and NO to enhance immunosuppression and promots tumor growth.	(56)
Lnc-C/EBPβ	–	MDSCs	Lnc-C/EBPβ suppresses the immunosuppressive capacity of MDSCs by suppressing the expression of NO and ROS.	(57)
LncRNA Pvt1	–	MDSCs	LncRNA Pvt1 knockdown suppresses G-MDSC-mediated immunosuppression *in vitro* by decreasing the level of ROS and ARG1, and delays tumor progression in tumor-bearing mice.	(61)
Lnc-Tim3	Hepatocellular carcinoma	CD8^+^ T cells	Lnc-Tim3 binds to Tim-3 and induces nuclear translocation of Bat3 in hepatocellular carcinoma, which compromises anti-tumor immunity by promoting CD8 + T cell exhaustion.	(64)
Lnc-sox5	Colorectal cancer	CD8^+^ T cells	Lnc-sox5 regulates the CD8^+^T cells infiltration and cytotoxicity through modulating the expression of IDO, further affecting the progression of colorectal cancer.	(65)
Lnc-EGFR	Hepatocellular carcinoma	Tregs	Lnc-EGFR specially binds to EGFR, enhances AP-1/NF-AT1/Foxp3 signaling, leading to Treg differentiation, and hepatocellular carcinoma progression	(68)
LncRNA SNHG1	Breast cancer	Tregs	Knockdown of lncRNA SNHG1 suppresses Treg differentiation by increasing the expression of miR-448 and reducing level of IDO, further alleviating the immune escape in breast cancer.	(69)
Flicr	–	Tregs	Flicr modulates the expression of Foxp3 and enhances the immunosuppressive function of Tregs.	(70)

*CAFs, cancer-associated fibroblasts; TAMs, tumor-associated macrophages; MDSCs, myeloid-derived suppressor cells; Tregs, regulatory T cells*.

## Exosomal LncRNAs-mediated Direct Communication between Tumor Cells and the TME

**Figure 2:**
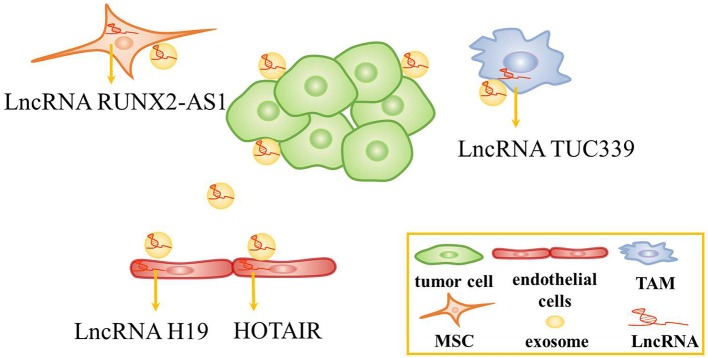
The role of exosomes in the tumor microenvironment. Tumor cells-derived exosomal lncRNAs are transferred to endothelial cells, tumor-associated macrophage (TAMs), and mesenchymal stem cells (MSCs) to alter the function of these cells.

Exosomes are critical mediators in intercellular communication through carrying intracellular components including DNA, RNA and protein to the recipient cells (71, 72). Tumor-derived exosomes can be used to change the TME, affect tumor cell proliferation, angiogenesis, and so on (73–75). In recent years, the role of exosomal lncRNAs in the TME has garnered increasing attention (28, 76). Several studies have shown that tumor cell-derived exosomal lncRNAs could affect the function of stromal cells in the TME (Figure 2).

Recent studies have revealed that tumor cell-derived exosomal lncRNAs are conveyed to endothelial cells to affect angiogenesis. For instance, lncRNA H19 was found to be highly expressed in CD90+ liver cancer cells. Interestingly, lncRNA H19, which was packed inside exosomes secreted by CD90+ liver cancer cells, was conveyed to and internalized by endothelial cells. Then, lncRNA H19 promoted angiogenesis by increasing VEGF production and release (77). Similarly, HOTAIR was abundant and was packed inside exosomes in glioma cells. Exosomal HOTAIR was transferred to endothelial cells, stimulating angiogenesis by upregulating the expression of VEGFA (78).

In addition, exosomal lncRNAs has been found to be transferred to macrophages and mesenchymal stem cells to alter the function of these cells. In HCC, TUC339 was identified as a kind of lncRNA abundant in tumor-derived exosomes and could be transferred from tumor cells to macrophages. Then, exosomal lncRNA TUC339 regulated macrophage cytokine production, M1/M2 polarization and phagocytosis, while the regulation mechanism needs further investigation (79). Besides, lncRNA RUNX2-AS1 could be packed inside exosomes and transmitted to mesenchymal stem cells. It might suppress the osteogenic differentiation of mesenchymal stem cells by modulating the expression of RUNX2 in multiple myeloma (80). Taken together, several studies revealed that tumor cells-derived exosomes could regulate the TME and affect tumor progression. Thus, tumor cells-derived exosomes containing lncRNAs might be served as biomarkers and therapeutic targets.

## LncRNAs as Potential Targets for Therapy

Numerous lncRNAs are aberrantly expressed in different cancer types, and their expression levels are associated with the initiation and progression of tumors (81). Recent and ongoing studies have improved our understanding of the role of lncRNAs in tumor biology (82). LncRNAs are emerging as novel molecules involved in tumor progression and are acted as promising biomarkers and therapeutic targets in cancer (83–85).

**Figure 3:**
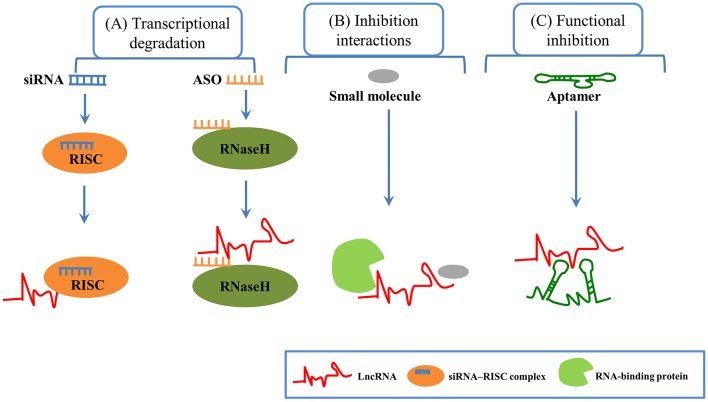
LncRNA-based therapeutic methods in cancer. **(A)** Transcriptional degradation: Short interfering RNAs (siRNAs), which are double-stranded RNAs, can degrade target RNAs through RISC (RNA-induced silencing complex). Antisense oligonucleotides (ASOs), which are single-stranded oligonucleotide sequence, offer specific complementarity, and Rnase H-mediated degradation of target lncRNA. **(B)** Inhibition interactions: Small molecules can inhibit the interaction between lncRNAs and protein. **(C)** Functional inhibition: Aptamers (short RNA or DNA oligonucleotides with 3-dimensional structure) can bind to their target lncRNAs at specific structural regions.

Due to their physiological and pathological roles in cancer, lncRNAs should be considered as candidates for biomarkers in cancer diagnostics and prognoses (29, 86, 87). For example, serum lncRNA HOTAIR was significantly higher in glioblastoma multiforme patients than in normal controls, which can be served as a novel diagnostic and prognostic biomarker in this disease (88). Similarly, exosomal lncRNA UEGC1 in the plasma was remarkably upregulated in early gastric cancer, indicating it might be a promising biomarker for early gastric cancer diagnosis (89). In a study of 246 subjects including 126 gastric cancer patients and 120 healthy controls, serum exosomal lncRNA HOTTIP was identified as a potential biomarker for diagnosis and prognosis in gastric cancer (90). LncRNAs are also emerging as therapeutic targets for cancer (91). Several features of lncRNAs need to be considered to support lncRNAs as therapeutic targets. First, lncRNAs structural complexity and participation in multicomponent complexes affords few potential targetable key residues to regulate structure-based interactions. Second, lncRNAs can play critical regulatory roles in gene expression and their expression levels are often lower than protein-coding genes. Third, lncRNAs are expressed in a tissue or cell-type specific manner, making them potential efficacious targets for tumor therapy. In addition, lncRNAs may also participate in cell-to-cell communication (84, 86, 92). LncRNAs can be targeted by multiple approaches, such as antisense oligonucleotides (ASOs), short hairpin RNAs (shRNAs), short interfering RNAs (siRNAs), aptamers, and small molecule inhibitor (Figure 3). ASOs also have already shown success in modulating coding genes involved in different kinds of solid tumors and other disease (93–96). ASOs are emerging as a potential therapeutic approach for targeting cancer-associated lncRNAs (97). For example, in the MMTV-PyMT mouse mammary carcinoma model, MALAT1 could promote tumor growth and metastasis. Then, in this model, the application of ASOs against MALAT1 resulted in slower tumor proliferation and a reduction in metastasis (98). Moreover, in a mouse xenograft model, MALAT1 ASOs was effective in suppressing lung cancer spreading (99). Thus, MALAT1 may be a potential therapeutic target, and MALAT1 ASOs may be a promising therapy approach for several types of cancer, but further assessment will be needed. A great number of studies have shown that siRNAs are used to against their target mRNAs for different disorders including cancer and metabolic disorders (100, 101). A few lncRNAs were silenced using siRNAs in cell lines (100, 102). However, preclinical studies using siRNAs/shRNAs to target lncRNAs were very limited. In human breast cancer cell lines, siRNA-mediated downregulation of HOTAIR inhibited cancer cell viability and matrix invasion (103). In human prostate cancer cell lines, siRNAs directed against MALAT led to suppression of cancer cell growth, migration and invasion (104). Moreover, subcutaneous injection of gastric cancer cell lines transfected with HOTAIR shRNA suppressed engraftment efficiency in nude mice (105). Aptamers can specifically bind to their target lncRNAs depending on the 3-dimensional shape of the lncRNA structures. Several reports have demonstrated promising effects of aptamers to modulate RNA functions, thus aptamers may be potential therapeutic agents to target lncRNAs. For example, Ayatollahi et al. demonstrated the aptamer-targeted Bcl-xL shRNAs delivery into lung cancer cells using alkyl modified PAMAM dendrimers (106). Small molecule inhibitors targeting a unique triple-helical structural element in lncRNAs (such as MALAT1 and NEAT1) are likely to destabilize the transcript to confer a therapeutic effect, although this needs further exploration (107–110).

## Conclusion

LncRNAs are multifunctional molecules that play critical roles in various biological processes, and dysregulated lncRNAs are often associated with a variety of pathophysiological conditions, such as cancer. We reviewed the recently published studies involving of lncRNAs in the TME. Emerging evidence indicates that lncRNAs play critical roles in modulating the TME and tumor progression. In addition, we described the crosstalk of lncRNAs between immune cells, CAFs, endothelial cells, and tumor cells in the TME and the promise of lncRNAs as tumor diagnostic markers and therapeutic targets.

Finally, tumor cells are thought to produce lncRNA-containing exosomes and tumor-derived exosomal lncRNAs may mediate direct communication between tumor cells and the TME, as illustrated in this review. Further research is still required to better understand the role of lncRNAs in the TME. In the future, lncRNAs may be used in strategies for early cancer detection, monitoring treatment responses and targeted cancer therapy.

## Author Contributions

DC and LW were the major contributors in writing the manuscript. TL and JT performed the literature search. HL and QW revised the manuscript. All authors read and approved the final manuscript.

### Conflict of Interest Statement

The authors declare that the research was conducted in the absence of any commercial or financial relationships that could be construed as a potential conflict of interest.
